# Evaluation of 17β-estradiol effects in mesenteric injury induced by occlusion of proximal descending aorta in male rats

**DOI:** 10.1186/1749-8090-10-S1-A163

**Published:** 2015-12-16

**Authors:** Paulo Thales Rocha de Sousa, Ana Cristina Breithaupt Faloppa, Cristiano de Jesus Correia, Laura Menegat, Raifd Restivo Simão, Roberto Armstrong, Sueli Gomes Ferreira, Alfredo Inácio Fiorelli, Luiz Felipe Pinho Moreira, Paulina Sannomiya

**Affiliations:** 1 Department of Cardiopneumology, Heart Institute (InCor) - University of Sao Paulo Medical School, Sao Paulo, 05403-000, Brazil

## Background/Introduction

Acute mesenteric ischemia is a life-threatening emergency with overall mortality ranging from 60% to 80%, and this survival rate has not improved substantially in recent decades. In surgical aortic reconstruction, occlusion of the aorta affects various organs through the ischemia reperfusion injury. Among these organs, the intestine is probably the most affected one. Several studies have proposed that oestradiol has a beneficial effect in the course of the inflammatory lesion.

## Aims/Objectives

This study aims to investigate the role of 17β-estradiol on mesenteric microcirculation after the occlusion of the descending aorta in male rats.

## Method

Male Wistar rats underwent mesenteric ischaemia by placing a Fogarty catheter^® ^in the aorta, that remained occluded during 15 minutes, followed by reperfusion up to 2 hours. Rats were divided into four groups: (1) rats that underwent surgical manipulation only (SHAM, n = 9); (2) rats submitted to ischaemia-reperfusion injury (I/R, n = 11); (3) rats treated with 17β-estradiol (E2, 280 μg/kg, iv) 30 minutes before I/R (pre-E2 I/R, n = 16); (4) rats treated with 17β-estradiol (E2, 280 μg/kg, iv) 15 minutes after the ischaemia induction (post-E2 I/R, n = 16)). Mesenteric perfusion was measured by intravital microscopy. Expression of eNOS was evaluated by immunohistochemistry and RT-PCR.

## Results

There was 40% decrease in the number of perfused small vessels (<30 μm diameter) in the group I/R compared to SHAM (p = 0.0386) associated with a reduction on endothelial nitric oxide synthase (eNOS) expression (p = 0,0126). The pre-E2 I/R treatment improved mesenteric perfusion (p = 0.0540) and eNOS expression (p < 0.0001) to levels attained in SHAM rats. The post-E2 I/R treatment normalised eNOS expression to reference levels. There were no differences in eNOS gene expression amongst groups.

## Discussion/Conclusion

Data presented suggest that either pre-treatment or post-treatment with 17β-estradiol enhances expression of eNOS on endothelial microvessels, improving mesenteric perfusion. 17β-estradiol treatment may be considered as an alternative to prevent major organs injury induced by aortic surgical procedures. Financial Support: 2013/02563-2 FAPESP

